# Use of Charlson Comorbidity Index and Nomogram to Predict Mortality in Elderly Patients with Late-Life Schizophrenia

**DOI:** 10.3390/healthcare9070783

**Published:** 2021-06-22

**Authors:** Mei-Chi Hsu, Shang-Chi Lee, Wen-Chen Ouyang

**Affiliations:** 1 Department of Nursing, I-Shou University, Kaohsiung 82445, Taiwan; hsu88@isu.edu.tw; 2 Department of Public Health, College of Medicine, National Cheng Kung University, Tainan 701, Taiwan; t88094022@gs.ncku.edu.tw; 3 Department of Geriatric Psychiatry, Jianan Psychiatric Center, Ministry of Health and Welfare, Tainan 71742, Taiwan; 4 Department of Nursing, Shu-Zen Junior College of Medicine and Management, Kaohsiung 82144, Taiwan; 5 Department of Psychiatry, College of Medicine, Kaohsiung Medical University, Kaohsiung 80708, Taiwan

**Keywords:** late-life schizophrenia, Charlson comorbidity index, mortality, nomogram, metabolic syndrome

## Abstract

*Objectives:* Comorbid illness burden signifies a poor prognosis in schizophrenia. The aims of this study were to estimate the severity of comorbidities in elderly patients with schizophrenia, determine risk factors associated with mortality, and establish a reliable nomogram for predicting 1-, 3- and 5-year mortality and survival. *Methods:* This population-based study rigorously selected schizophrenia patients (≥65 years) having their first admission due to schizophrenia during the study period (2000–2013). Comorbidity was scored using the updated Charlson Comorbidity Index (CCI). *Results:* This study comprised 3827 subjects. The mean stay of first admission due to schizophrenia was 26 days. Mean numbers of schizophrenia and non-schizophrenia-related hospitalization (not including the first admission) were 1.80 and 3.58, respectively. Mean ages at death were 73.50, 82.14 and 89.32 years old, and the mean times from first admission to death were 4.24, 3.33, and 1.87 years in three different age groups, respectively. Nearly 30% were diagnosed with ≥3 comorbidities. The most frequent comorbidities were dementia, chronic pulmonary disease and diabetes. The estimated 1-, 3- and 5-year survival rates were 90%, 70%, and 64%, respectively. Schizophrenia patients with comorbid diseases are at increased risk of hospitalization and mortality (*p* < 0.05). *Conclusion:* The nomogram, composed of age, sex, the severity of comorbidity burden, and working type could be applied to predict mortality risk in the extremely fragile patients.

## 1. Introduction

Schizophrenia is one of the most severe chronic debilitating mental diseases. Patients with schizophrenia have a higher premature mortality rate than the general population [1,2]. It is known that life expectancies of schizophrenia patients in comparison with the general population are 10–25 years shorter [3,4]. Similarly, life expectancy of schizophrenia patients in Taiwan is shorter than the public, and their higher death rates are due most probably to physical comorbidities [5,6]. Despite evidence having shown associations between proper management of schizophrenia and better outcomes, poor treatment adherence in patients with resistant schizophrenia leads to impaired social and cognitive functioning, psychiatric hospitalizations and increased treatment costs [7]. Mortality rates also remain at an inappropriately high level [3,6].

The “elderly” has been defined as having a chronological age of 65 years or older [8,9]. The incidence of schizophrenia after the age of 65 years is 7.5 per 100,000 person-years [10]. A study is presented in support of the treatment, policy, and research needs and perspectives on schizophrenia in later life [11]. The authors indicated that older persons (aged 55 years and older) with schizophrenia have been largely neglected due to the lack of studies devoted to this population, with only about 1% of schizophrenia literature focused on this demographic, and an absence of relevant guidelines [11].

Late-life schizophrenia comprised three distinct groups: persons with early-onset schizophrenia (EOS) who have grown old; late-onset schizophrenia (LOS, after age 40); and very-late-onset schizophrenia-like psychosis (VLOSLP, after age 60) [12,13]. A Dutch study found a 1-year prevalence rate of 0.55% for schizophrenia among persons older than 59 years, of whom 64% had EOS, while 36% had LOS or VLOSLP in late life [11]. Thus, the 1-year prevalence of EOS and LOS/VLOSLP was calculated as 0.35% and 0.20%, respectively. Mortality rates in VLOSLP patients were higher than those in EOS patients, due mainly to excess physical comorbidities [11,14].

Comorbidities, exerting an important impact on mortality and survival, constitute one of the important confounding factors that must be well understood prior to properly and adequately carrying out the analysis of mortality risk. Importantly, the implementation of comorbidity scores in studies assessing prognosis is critical for the aging population. Indeed, medical comorbidity in older people with schizophrenia is associated with numerous negative outcomes. Further, comorbid diseases are important factors that are predictive of health [15]. Medical comorbidity is also associated with poorer neurocognitive functioning [16]. Declines in functional and cognitive status as well as increased mortality rates are consequences in elderly patients who have medical comorbidity. The risk of mortality in patients with schizophrenia is two to three times greater than that in the general population [11]. The health challenges of this population could have contributed to the increase in overall mortality. Further, in this ageing schizophrenia population, neurobiology of LOS and VLOSLP is different from those of EOS, including coinciding atrophy and hypo-metabolism of the frontal, temporal and subcortical areas [17,18]. The neuropsychology and long-term outcome of LOS and VLOSLP as a precursor of cognitive decline is also an important concern [11,17,19,20]. This population may encounter these issues both in theory and in practice; for example, the end of a prodromal phase, and the significant signs of cognitive decline and poor physical functioning, are comorbid with undetected delirium.

In comparison with the general public, the prevalence of physical comorbidities in patients with schizophrenia is high, with odds ratios varying from 2.62 to 7.54 [21]. Approximately two-thirds of the premature mortality in this population is attributable to physical comorbidities, such as dyslipidemia, coronary artery disease, cancer, and dementia [22,23]. Patients with schizophrenia since illness onset or even in antipsychotic-naive patients have an increased risk of comorbidities such as diabetes [24].

Schizophrenia may be a risk factor for various comorbid diseases. Medical problems may also be caused by factors such as cognitive and behavioral impairments associated with schizophrenia itself, or the adverse effects related to drugs used for treatment. For example, clozapine is one of common drugs used for treatment-resistant older patients with schizophrenia [2,7]. Clozapine is associated with the development of a number of adverse effects such as myocarditis, weight gain, and, later on, metabolic syndrome [7]. High vulnerability to adverse reactions to clozapine should be noted in this population despite its proven efficacy [7]. Thus, a well-targeted comprehensive geriatric assessment is required when clozapine treatment is prescribed in the elderly [7]. In addition, medical comorbidity appears to be apparently higher in schizophrenia only or dual diagnosis, such as both alcohol dependence and schizophrenia [16,25]. Patients who have poor physical condition with comorbidities are also at increased risks of hospitalization.

As comorbidities in older patients with schizophrenia account for a substantial burden to health services, the ability to characterize the comorbidity burden of this population is of great importance in psychiatric care. Many different methods have been used to characterize the comorbidity in patients with schizophrenia. For example, the Elixhauser comorbidity index (ECI) [21], the Selim comorbidity index [26], and the Charlson comorbidity index (CCI) [27,28] have been used to account for comorbidity in different populations. Earlier, the CCI has also been used for predicting mortality in patients with schizophrenia [29]. The CCI was superior in predicting mortality and, in particular, considers the severity of conditions. This method may be a useful approach to risk or comorbidity adjustment and could reduce potential confounding in health services research. The CCI has also been applied in predicting the outcome of mortality among persons with late-life schizophrenia.

In recent years, many prognostic models based on clinical and medication treatment variables have been developed to predict clinical and treatment outcomes. However, there is no study reported to date, using a large, nationally representative sample, to assess the medical comorbidity in the first admission of elderly patients with late-life schizophrenia. There is also no validated method to ascertain the severity of the comorbidity burden for risk-adjustment in this population using nationally representative health care databases. Therefore, determining the severity of comorbidity burden for mortality and survival is imperative for the continuous improvement in prognosis of elderly patients with late-life schizophrenia.

The nomogram is an important element of the medical decision-making model. This model has been widely used as a prognostic tool for different diseases. This model can also be useful in developing an effective health care intervention. To date, a nomogram model that has adequate power and ability to predict mortality in elderly patients with late-life schizophrenia has not been developed.

The purposes of this study were as follows: (1) to estimate the severity of comorbidities in elderly patients with schizophrenia (aged 65 years or older) who had their first hospitalization after diagnosis of schizophrenia; (2) to assess the association between different risk factors (e.g., age, gender) and mortality; and (3) to establish a novel and reliable nomogram model for predicting 1-, 3- and 5-year mortality and survival with external validation for this population. Hazard of death was also calculated.

## 2. Materials and Methods

### 2.1. Data Sources and Study Population

A retrospective cohort study, making use of National Health Insurance Research Database (NHIRD), was carried out. The database consists of medical records of all 23 million citizens and foreigners who reside in Taiwan, including ICD-coded diagnoses (associated events and comorbidities). Based on this database, we were also able to carry out epidemiological studies. The protocol of this research was approved by the Institutional Review Board of hospitals. All personal information is encrypted for privacy protection.

**Figure 1:**
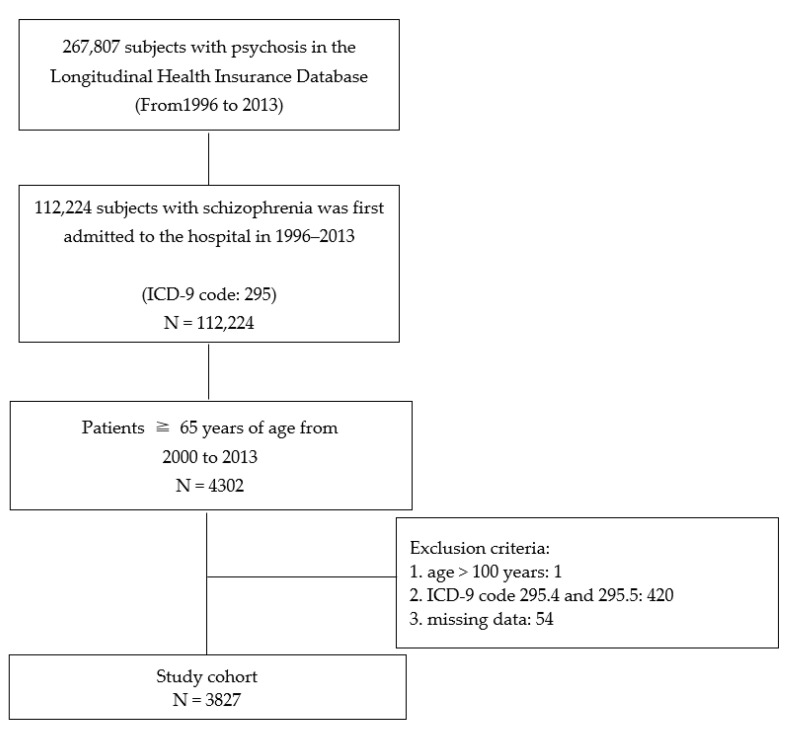
Flowchart of identification and enrolment of the study subjects.

Subjects were included prospectively and followed if they: (1) were aged ≥65 years; (2) had a major psychiatric diagnosis of schizophrenia, using the ICD-9 coding system, by psychiatrists; and (3) had their first hospitalization because of schizophrenia after 65 years of age between January 2000 and December 2013. The index hospitalization was defined as the first hospitalization during this study period. Each subject was identified by all diagnoses in all hospital admissions. Subjects were excluded if at least one of the following had occurred: (1) a hospitalization history because of schizophrenia prior to 65 years of age; (2) ICD-9 code 295.4 and 295.5 due to poor diagnostic stability [30,31]; (3) missing data; and (4) >100 years of age. The flow chart outlining the inclusion and exclusion of patients is shown in Figure 1.

The selection of schizophrenia patients with first-time hospitalization after 65 years of age is important. First-time admission to a psychiatric ward for acute psychosis can be a useful indicator of improvement of illness severity, particularly for this ageing schizophrenia population. They may require more healthcare attention to address their mental and physical health conditions and comorbidity because of their unique health challenges. The first hospitalization in this population also represents the first comprehensive contact with psychiatric services, and can, therefore, be considered a good indicator of mental and physical condition. More in-depth evaluation is required before establishing appropriate policies for health care in this population.

### 2.2. Outcomes

The main outcomes were 1-, 3-, and 5- year overall mortality, mean age at death (years) and time from 1st admission to death (years).

### 2.3. Confounding Variables

The following potential confounding sociodemographic and clinical variables were assessed: age (65–74, 75–84 and 85+), sex, working type, hospital locations where patients seek medical attention (north, central, south, east, and offshore), hospital level (medical center, metropolitan hospital, local community hospital, physician clinics) and Charlson comorbidity index (CCI).

Working types were grouped into 3 categories: high (e.g., government official, military officers and teachers), medium (non-government official/military officers/teachers), and low (e.g., low-income households, veterans, and casual/seasonal employment). This study also includes the number of hospitalizations (related or non-related to schizophrenia), and length of hospital stay of the first admission due to schizophrenia.

Quan et al. [32] have updated the conditions and weights included in the CCI, simplified the score and validated an updated CCI for the prediction of mortality. The enhanced version of CCI comprises 17 comorbid conditions including myocardial infarction, congestive heart failure, peripheral vascular disease, cerebrovascular disease, dementia, chronic pulmonary disease, rheumatic disease, peptic ulcer disease, mild liver disease, diabetes without chronic complication, diabetes with chronic complication, hemiplegia or paraplegia, renal disease, any malignancy (including lymphoma and leukemia, but not including malignant neoplasm of skin), moderate or severe liver disease, metastatic solid tumor, and AIDS/HIV. This enhanced CCI system has been used extensively as a measure of comorbid illness severity and the prognosis of patients. The comorbidity was identified at the date of enrollment.

### 2.4. Statistical Analysis

Statistical Analysis System (version 9.4, SAS Institute, Inc. Cary, NC, USA) and R statistical software (version 3.0.2, R Foundation for Statistical Computing, Vienna, Austria) were used. The precision and accuracy of the statistical information retrieved from the database was ratified by two statisticians. Individuals were followed until they passed away, or the end of the study period—whichever came first. All clinical outcomes after index hospitalization were recorded over a 14-year period. Categorical variables were expressed as n (%), while continuous variables were indicated as means ± standard deviation (SD) or medians (interquartile interval). Comparisons of qualitative variables between groups were conducted using the chi-square test or Fisher exact test under the conditions of application. Comparison between the age groups were performed using analysis of variance (ANOVA). Our intention for including estimation of the extra 5-year age interval for both diabetes and dementia was to allow further examination of the importance of age in this population.

The sensitivity analysis was performed by applying a Cox proportional hazards regression model. Hazard ratios (HRs) with 95% confidence level (CIs) were calculated after adjustment for potential confounders, such as age and CCI scores.

To construct the nomogram, multivariate regression analysis was used to select the significant predictors of mortality that included variables with *p* values < 0.05 in univariate analysis. The consistency index (C-index), the calibration curve (1000 bootstrap resamples) and the receiver operating characteristic (ROC) curve were used to evaluate the predictive performance of the nomogram. The calibration of the nomogram was tested using a calibration plot, which showed the degree of fit between actual and nomogram-predicted mortality. The *p* value < 0.05 was defined as statistically significant.

## 3. Results

The study comprised 3827 elderly patients with schizophrenia. All demographic and clinical characteristics were shown in Table 1. The mean age of the patients was 72.89 (±6.37) years. Most patients (n = 2433, 63.57%) were in the 65–74 age group. Only 5.96% (n = 228) were found in the 85+ age group. There were more males than females in all age groups, except for the 65–74 age group. Most patients (n = 2253, 58.88%) were from low-income households, were veterans, or in casual/seasonal employment; this trend was more pronounced in males (n = 1380, 68.75%).

Most hospitals where patients use medical and health services were located in the North (n = 1279, 33.42%). More males used medical and health services in the North (n = 528, 26.31%) and East (n = 518, 25.81%) whereas females were mainly in the North (41.26%). Most patients, particularly males, used health services in local community hospitals (n = 1695, 44.29%).

### 3.1. Hospitalization

The mean days of first admission due to schizophrenia was 26 days; this period was longer in females than males (28 vs. 22), but shorter in the 85+ age group. The mean for the follow-up year was 4.11; this was shorter in males (3.58) and the 85+ age group (2.14). The schizophrenia-related and non-schizophrenia-related mean numbers of hospitalizations (not including the first admission) were 1.80 (±3.37) and 3.58 (±5.48), respectively. Significant age and sex differences were found in these variables (*p* < 0.05).

### 3.2. Deaths

A total of 49.62% (n = 1899/3827) of patients were deceased, which comprised 64.13% (n = 1218) of male and 35.86% (n = 681) of female elderly patients (Table 1). Mean ages at death were 73.50, 82.14 and 89.32 years old in the three age groups, respectively. Mean age at death was 78.77 for males, and 77.41 for females. Among different age groups, 40.36% in the 65–74 age group, 64.75% in the 75–84 age group, and 71.05% in the 85+ age group passed away throughout the study period.

The average time lengths between the first admission and their death in different age groups were 4.24, 3.33, and 1.87 years, respectively. The length of hospital stay was 111.49 days for male patients and 86.90 days for female patients. The numbers of hospitalizations were 6.18 and 4.50 for males and female patients.

### 3.3. Comorbidities

Table 2 and Table 3 show the CCI scores and the prevalence of each CCI in the study sample. It should be noted that some patients were diagnosed with more than one comorbidity. The average number of comorbid medical conditions was 1.82 (±1.98). Men were likely to have experienced more comorbidities than women (2.04 vs. 1.59). Dementia was the most frequent comorbidity across all age groups. The top 2–5 most common comorbidities across the age groups were similar, namely diabetes, peptic ulcer disease, chronic pulmonary disease, and cerebrovascular disease.

Nearly 30% (n = 1090) were diagnosed with ≥3 medical conditions. When the cut-off point was set at 3, 71.52% of the patients has low CCI scores (<3), whereas 28.48% had high CCI scores (≥3).

The most frequent medical conditions were dementia (33.81%), chronic pulmonary disease (25.42%) and diabetes (22.29%). The top five most prevalent comorbidities were similar among different age groups. Particularly, dementia (68.42%) and chronic pulmonary disease (48.25%) rose rapidly in the 85+ age group.

### 3.4. Adjusted Models of Overall Mortality

Results from the multivariate Cox proportional hazards analysis, which was used to compare the proportion of patients who developed the primary outcomes, show that age, sex, CCIs, and working type were all potential predictors for mortality (*p* < 0.01; Table 4). For example, males had a HR that was 1.66 times higher than that of females. The CCI score was also a significant and independent risk factor for mortality. The CCI score in elderly patients with schizophrenia was significantly and positively correlated with mortality (HR, 1.14; *p* < 0.0001). In other words, the overall survival for patients with a higher score was significantly worse than that for patients with a lower score.

**Figure 2:**
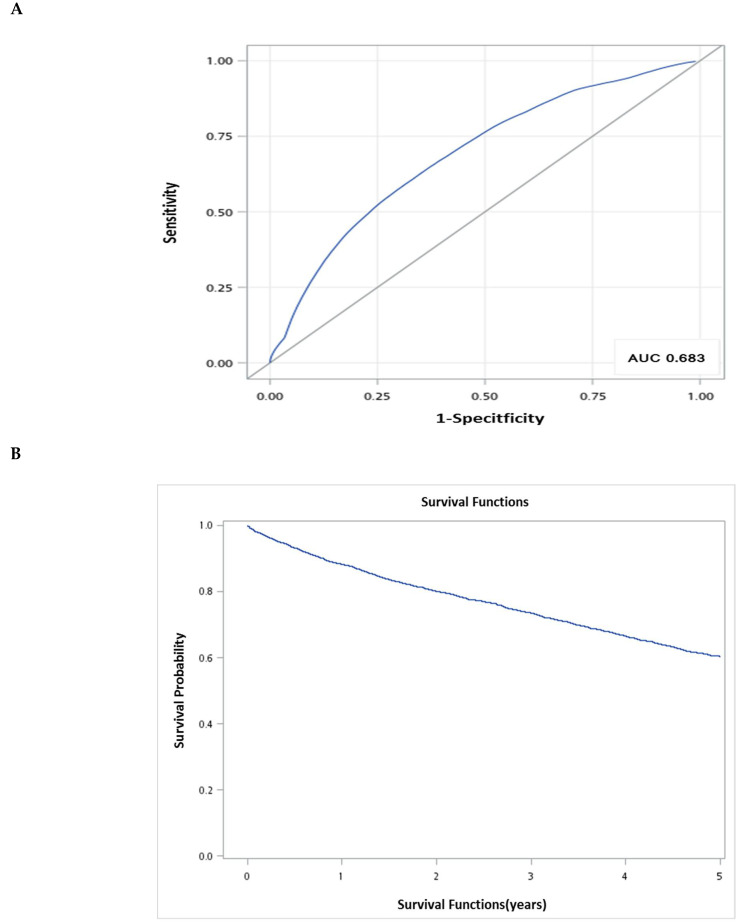
(**A**) Receiver operating characteristic curve (ROC curve) of mortality prediction models at 5-year follow up, including sex, age, and CCI (continuous variable). (**B**) 5-year overall survival rate following schizophrenia.

The predictive accuracy of Cox regression output was characterized by time-dependent sensitivity, specificity, and associated receiver operating characteristic (ROC) curves, where survival time was the outcome. The areas under the ROC curve (AUC) were calculated to be 0.683, indicating that this is a good model for predicting mortality (Figure 2A). The bootstrap corrected AUC for the final model was 71%.

The overall survival curve using the Kaplan–Meier method with different survival times (times-to-event) is shown in Figure 2B. The 1-, 3- and 5-year survival rates were estmated to be 90%, 70%, and 64%, respectively.

**Figure 3:**
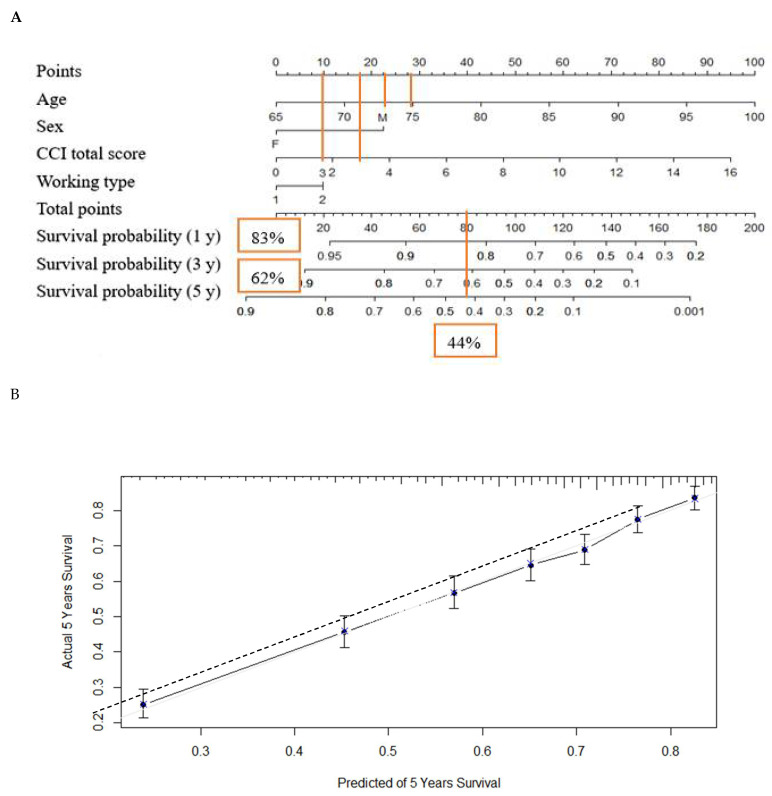
(**A**) Nomogram predicting 1-, 3- and 5-year survival probabilities. The sum of the scores for each variable was plotted on the total points axis (the horizontal line at the top,) and the estimated probabilities of survival at 1, 3 and 5 years were obtained by drawing a line vertically from the plotted total point axis straight to the survival axis. (**B**) Calibration plots comparing nomogram-predicted (- - - - -) and actual (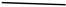) survival probabilities at 5 years.

Based on the sum of the assigned number of points for each predictor in the nomogram, the results in Figure 3A show that the higher the total points, the greater the associated risk of mortality. For example, a patient who was 75 years old (29 points) and male (22.5 points), with CCI score of 3 (18.5 points) and a work type of 2 (10 points), would have a total of 80 points. The 80 total points predicted a 1-year survival rate of about 83%, a 3-year survival rate of 62%, and a 5-year survival rate of around 44%.

Figure 3B shows the calibration plot comparing the prediction of survival probabilities between actual observation and the nomogram. The dashed diagonal line represents a perfect concordance between the observed and predicted survival probabilities. The calibration plot from this study revealed a good predictive accuracy of the nomogram, and a nearly perfect concordance between the predicted and observed proportion of surviving patients.

## 4. Discussion

This nationwide population-based study, as far as we are aware, is the first and largest study focusing and investigating the elevated risk of comorbidities conferred by elderly patients with schizophrenia, and specifically, those having their first hospitalization because of schizophrenia during the study period. Moreover, this study is the first to evaluate the efficacy of using CCI to assess medical comorbidities in elderly individuals with schizophrenia in Taiwan, as potential predictive factors of mortality. We found that age, sex and CCI scores in elderly patients with schizophrenia were significantly associated with risk of mortality. This population-based study focusing on this elderly population provides detailed epidemiological facts on patients and allows up to 3 years of follow-up.

One of the strengths of this study is the use of a nomogram for the prediction of overall mortality. We established a precise and reliable nomogram by converting the total score into a continuum of individual scores through a logarithmic formula. When this nomogram was used to predict the probability, from 0.1% to 95%, of mortality at 1, 3 and 5 years for elderly patients with schizophrenia following their first hospitalization due to schizophrenia, the results demonstrated very good discrimination and accuracy in predicting mortality in this population, which may aid patient management.

A recent study [33] indicated that the major cost in elderly patients with schizophrenia is the treatment of the patients’ non-psychiatric medical illnesses. The important finding of this study is that nearly 70% (n = 2645) of elderly patients with schizophrenia had at least one comorbid medical condition. The comorbidity proportion in schizophrenia reported in this study is similar to that of Batki et al. [25], but much higher than the proportions reported in two other studies [16,34]. As shown in this study, about half (49.56%) of those in the 85+ group had CCI scores ≥ 3. Advanced age and high CCI scores have previously been shown to be related to poor clinical outcomes in patients. Consistent with a previous report [35], our study has also shown increased mortality rates in males. Even worse, patients with severe mental disorders may receive limited medical services or lower quality medical care [35]. Therefore, it is important that elderly participants with high levels of comorbid diseases be well informed of their high clinical risk and advised to look for suitable medical care.

Diabetes was the third most common comorbidity in elderly patients with schizophrenia. In the present study, the rate of diabetes—affecting nearly one fifth of this population—was significantly higher than that found in other studies (22.29 vs. 10–11%) [16,25,36]. Except for inpatients with schizophrenia who were older than 70 years, the prevalence of diabetes among these patients was similar to levels reported previously by other Taiwanese studies [36,37]. The above results may support the hypothesis that schizophrenia and diabetes may share the same genetic mechanisms [38]. However, the onset age of schizophrenia is much earlier than that of type 2 diabetes [39].

Recently, a study has demonstrated that schizophrenia patients have greater risks of rehospitalization due to aberrant glucose metabolism, such as hypoglycemia (adjusted odds ratio 3.21), hyperglycemia (7.01), ketoacidosis (2.01) and coma (3.17) [40]. Diabetes has deleterious effects on neurological function, which were mediated by several mechanisms such as endothelial damage and intracellular acidosis, all of which may lead to severe neurological deficit and higher risk of mortality or morbidity with dementia [41,42] in this population. Thus, patients with schizophrenia suffering concurrently with diabetes are at higher risk of hospitalization for complications and mortality.

Patients with psychotic disorders are less likely to be recommended testing of HbA_1c_, lipid profile, or microalbumin. These patients have high risk in hospitalization for diabetes complications and diabetes-related or all-cause mortality [40]. Poor diabetes control in schizophrenia could also be caused by unhealthy diet, physical inactivity, antipsychotics and impaired cognition [43]. Moreover, the patients with schizophrenia in our study were aged ≥ 65 years, had been initially hospitalized after 65 years of age because of schizophrenia, and were treated with standard care. It has been reported that recently developed antipsychotics, including third-generation antipsychotics, have a positive effect because treatments with antipsychotics improve glycemic control thorough the improvement of the psychotic symptoms in schizophrenia [44]. They also lead to improvements in adherence to diabetes treatment and patients’ quality of life. Furthermore, clozapine, an atypical antipsychotic, is effective in everyday clinical practice, relieves psychotic symptoms, and achieves a satisfactory quality of life [7,45].

In this study, dementia ranked first among the comorbid conditions, affecting one third of this elderly population (33.81%). Our finding is significantly higher than that reported in the previous Taiwanese studies (odds ratio = 4.2 vs. 1.8 in Sun et al. [46] and Lin, Chung, Chen, and Chi [47], respectively). Schizophrenia shows a further cognitive decline with aging. The diagnosis of dementia can sometimes be difficult to confirm and confused with various psychiatric disorders, particularly schizophrenia. Stability of diagnosis is important for validation. The findings of our study may provide an impetus for further exploration of the relationship between schizophrenia and dementia.

Differentiating schizophrenia and Alzheimer disease in older adults can be somewhat difficult, although there are distinguishing features between them. Both diseases share some similarities in symptoms and functions. For example, both diseases affect similar areas of the brain. Several hypotheses have been proposed that there exists a close relationship between schizophrenia and fronto-temporal lobar dementia [48], a common cerebral pathophysiology, or a common genetic risk of Alzheimer’s disease and schizophrenia [49], as well as an involvement of the dysregulation of the calcium signaling pathway in the developmental processes of both Alzheimer disease and schizophrenia [50].

It is not easy to differentiate between a diagnosis of VLOSLP and dementia with psychotic symptoms, which was one of the Behavioral and Psychological Symptoms of Dementia (BPSD) in the elderly. VLOSLP and dementia with psychotic symptoms both have similar symptomatology. However, the characteristic features of partition delusions and auditory hallucinations of human voices may be relatively more commonly seen in VLOSLP than Dementia with Lewy bodies (DLB) and Alzheimer’s-type dementia with psychosis [13,51]. The recurrent visual hallucinations exhibited in DLB are typically well formed and detailed, as shown in McKeith et al., 2017 [52], but this is less frequently seen in VLOSLP. The reduced learning and consolidation skills strongly explain a significant part of the clinically impaired performance commonly reported in Alzheimer’s-type dementia with psychotic symptoms compared with VLOSLP [18,51]. The prominent visuoconstructive deficits can be observed in DLB. Impairment of cognitive function appeared after psychotic symptoms in VLOSLP, but this cognitive impairment occurred ahead of psychotic symptoms in dementia with psychotic symptoms in longitudinal psychiatric history.

The 5-year stratified prevalence of dementia among elderly patients with schizophrenia in this study is also higher than those reported in a previous community-based study in Taiwan [46]. The age-specific prevalence rates for 65–69, 70–74, 75–79, 80–84 and >85 years were 18.76, 31.50, 42.74, 56.88 and 68.42%, which were much higher than the 3.40, 3.46, 7.19, 13.03 and 26.32%, respectively, reported in a study by Sun et al. [46]. Thus, our findings for this population do not seem to reflect the phenomena of “dementia incidence doubles every 5 years from ages 65 to 90 years” mentioned in other studies [46,53,54]. The possible explanations for the higher prevalence of dementia in elderly schizophrenia patients seen in this study may be pertinent to higher rates of concurrent comorbid diabetes, cardiovascular diseases, chronic lung diseases, and traumatic head injury, which is also supported by Lin et al. [47]. More importantly, patients with chronic pulmonary diseases such as pneumonia and chronic obstructive pulmonary (COPD) may have an increased risk of dementia. In this study, chronic pulmonary diseases have been found to be the second most common category of comorbidity (25.4%). As previously found in a study, increased risk of dementia may be seen in patients with chronic obstructive pulmonary disease [55]. Pneumonia is a life-threatening infection that is a significant risk factor for mortality in the elderly.

Earlier, Emil Kraepelin’s concept of “dementia praecox” included the assertion that schizophrenia, as a chronic, deteriorating psychotic disorder characterized by rapid cognitive disintegration, premature aging, and shorter life expectancy [3,56], may cause the rapid increase in prevalence of dementia. Moreover, first- and second-generation antipsychotics have been shown to increase the risk of cerebrovascular events and comorbid dementia in schizophrenia [47]. However, first-generation antipsychotics were first introduced in the 1950s, while Taiwan launched a National Health Insurance Program in 1995. The elderly schizophrenia patients whose onset year could be traced back to between 1950 and 1995 might not have benefited from the early intervention with first-generation antipsychotics. Thus, the timing of when antipsychotics were introduced, and Taiwan’s health insurance program was implemented, had major impacts on how patients, particularly this elderly population, were treated.

Taken together, schizophrenia patients with comorbid dementia represent a population that is particularly vulnerable to delirium, and somatic and psychological problems. It is conceivable that the hospitalization rates will be largely increased in schizophrenia patients who also have comorbid dementia regardless of whether these patients are also at risk of health crises due to other physical health-related factors. Treatment of the delirium/behavioral and psychological symptoms of dementia (BPSD) patients with psychotic symptoms in a similar manner to the treatment of late-onset schizophrenia patients should be avoided. When psychotic state and concurrent physical illness are seen in elderly patients, it is worth mentioning that clarification of the temporality between psychosis and medical condition is necessary before diagnosis of schizophrenia is made.

The present study has notable limitations. The inherent limitations of the retrospective NHIRD data might hamper the analyses in terms of the reflection of some clinical outcomes. The analysis could not reflect the detailed characteristics and risk factors of these conditions, particularly the severity of comorbid illnesses. The details about antipsychotic intake in patients were also not collected in this study. Moreover, patients with intellectual disability coded as 317–319 in ICD-9 might be considered to be excluded in order to eliminate the confounding effect [57].

## 5. Conclusions

We have meticulously selected from the population-based database, a large sample of elderly patients aged 65 years and older, having first hospitalization due to schizophrenia after 65 years of age. We established a precise and reliable nomogram with external validation. The nomogram was based on variables that can be easily extracted from the clinical records and used in a clinical setting. The nomogram, composed of age, sex, the severity of comorbidity burden, and working type, may predict mortality risk in extremely fragile patients.

The high health risks recognized in patients with schizophrenia stress the need for provision of efficient medical care. These patients are in need of greater attention, specifically in the area of comorbid diseases of schizophrenia. The findings of our study would necessitate that health professionals anticipate and consider the comprehensive geropsychiatric interview, assessment and diagnosis of this population. Persons with late-life schizophrenia should be provided with early possible strategies for prevention and promotion of mental health against comorbid dementia and other medical problems that may reduce the risk of developing dementia. Future studies are recommended to further develop a greater understanding of whether schizophrenia, comorbidity or other health problems are potentially modifiable risk factors or play a role in dementia.

## Figures and Tables

**Table 1:** Baseline demographics and clinical characteristics of the patient population (age-group and sex comparisons).

	Total (*n* = 3827)	Age Groups				Sex		
		65–74 (*n* = 2433) 63.57%	75–84 (*n* = 1166) 30.47%	85+ (*n* = 228) 5.96%	*p* Value	Male (*n* = 2007) 52.44%	Female (*n* = 1820) 47.56%	*p* Value
Age distribution								
Mean age (years)	72.89 ± 6.37					74.07 ± 6.70	71.60 ± 5.71	
No. of males (%)	2007	1124 (56.00)	716 (35.68)	167 (8.32)	<0.0001			
No. of Females (%)	1820	1309 (71.92)	450 (24.73)	61 (3.35)				
Number of hospitalization (except first admission)								
SZ-related	1.80 ± 3.37	2.04 ± 3.68	1.47 ± 2.79	0.95 ± 2.05	<0.0001	1.91 ± 3.71	1.69 ± 2.94	0.0423
Non-SZ-related	3.58 ± 5.48	3.29 ± 5.66	4.19 ± 5.26	3.49 ± 4.38	<0.0001	4.27 ± 6.08	2.81 ± 4.62	<0.0001
Hospital stay (days) of first admission due to schizophrenia								
Days	26.00	29.00	20.00	14	0.0613	22.00	28.00	0.0110
(Q1, Q3)	(9.00, 58.00)	(10.00, 60.00)	(8.00, 52.00)	(7.00, 41.50)		(8.00, 59.00)	(10.00, 56.00)	
Follow-up								
Year	4.11	6.71	4.43	2.14	<0.0001	3.58	4.70	<0.0001
(Q1, Q3)	(1.66, 7.35)	(3.05, 11.39)	(1.74, 8.39)	(0.75, 4.38)		(1.30, 6.75)	(2.23, 7.89)	
Working type								
Heavy labor	614 (16.04)	444 (18.25)	153 (13.12)	17 (7.46)	<0.0001	198 (9.87)	416 (22.86)	<0.0001
Moderate labor	960 (25.08)	698 (28.69)	229 (19.64)	33 (14.47)		429 (21.38)	531 (29.18)	
Light labor	2253 (58.88)	1291 (53.06)	784 (67.24)	178 (78.07)		1380 (68.75)	873 (47.97)	
Hospital location								
North	1279 (33.42)	859 (35.31)	363 (31.13)	57 (25.00)	<0.0001	528 (26.31)	751 (41.26)	<0.0001
Center	857 (22.39)	593 (24.37)	222 (19.04)	42 (18.42)		442 (22.02)	415 (22.80)	
South	1010 (26.39)	676 (27.78)	285 (24.44)	49 (21.49)		507 (25.26)	503 (27.64)	
East	664 (17.35)	297 (12.21)	287 (24.61)	80 (35.09)		518 (25.81)	146 (8.02)	
Offshore	17 (0.45)	8 (0.33)	9 (0.78)	-		12 (0.60)	5 (0.27)	
Hospital level								
Medical centers	608 (15.89)	414 (17.02)	160 (13.72)	34 (14.91)	<0.0001	215 (10.71)	393 (21.59)	<0.0001
Metropolitan hospitals	1513 (39.53)	1087 (44.68)	371 (31.82)	55 (24.12)		667 (33.23)	846 (46.48)	
Local community	1695 (44.29)	923 (37.94)	633 (54.29)	139 (60.96)		1121 (55.85)	574 (31.54)	
Physician clinics	11 ( 0.29)	9 (0.37)	2 (0.17)	-		4 (0.21)	7 (0.38)	
Death								
Number of deaths (from first admission of SZ)	1899	982	755	162		1218	681	
Mean age at death (years)		73.50 ± 4.32	82.14 ± 3.83	89.32 ± 2.94		78.77 ± 6.68	77.41 ± 6.54	
(Q1, Q3)		(70.00, 76.00)	(79.00, 85.00)	(87.00, 91.00)		(74.00, 84.00)	(72.00, 82.00)	
Time from 1st admission to death (years)		4.24 ± 3.19	3.33 ± 2.86	1.87 ± 1.71		3.41 ± 2.97	4.15 ± 3.12	
(Q1, Q3)		(1.46, 6.47)	(0.945, 4.95)	(0.44, 3.06)		(0.88, 5.20)	(1.46, 6.30)	
Days of hospitalization		106.80 ± 330.15	92.62 ± 258.63	61.75 ± 158.19		111.49 ± 332.89	86.90 ± 263.52	
(Q1, Q3)		(10.00, 60.00)	(8.00, 52.00)	(7.00, 41.50)		(8.00, 59.00)	(10.00, 56.00)	
Number of hospitalizations		5.33 ± 6.90	5.67 ± 5.91	4.44 ± 4.96		6.18 ± 7.19	4.50 ± 5.55	
(Q1, Q3)		(1.00, 7.00)	(2.00, 8.00)	(1.00, 6.00)		(2.00, 8.00)	(1.00. 6.00)	

Note. Data were presented as Mean ± SD, N (%), (Q1, Q3); * including unemployed people or those who had no fixed occupation.

**Table 2:** CCI scores and numbers of patients (%). Comparison of age group and sex.

CCI Scores	Total (n = 3827)	Age Group			Sex	
		65–74 (n = 2433)	75–84 (n = 1166)	85+ (n = 228)	Male (n = 2007)	Female (n = 1820)
0	1182 (30.89)	899 (36.95)	254 (21.78)	29 (12.72)	540 (26.91)	642 (35.27)
1	902 (23.57)	612 (25.15)	258 (22.13)	32 (14.04)	455 (22.67)	447 (24.56)
2	653 (17.06)	374 (15.37)	225 (19.30)	54 (23.68)	359 (17.89)	294 (16.15)
3	443 (11.58)	230 (9.45)	178 (15.27)	35 (15.35)	250 (12.46)	193 (10.60)
4	271 (7.08)	145 (5.96)	97 (8.32)	29 (12.72)	171 (8.52)	100 (5.49)
5	170 (4.44)	87 (3.58)	59 (5.06)	24 (10.53)	89 (4.43)	81 (4.45)
6	89 (2.33)	34 (1.40)	48 (4.12)	7 (3.07)	66 (3.29)	23 (1.26)
7	45 (1.18)	23 (0.95)	14 (1.20)	8 (3.51)	31 (1.54)	14 (0.77)
8	35 (0.91)	16 (0.66)	15 (1.29)	4 (1.75)	22 (1.10)	13 (0.71)
9	14 (0.37)	6 (0.25)	6 (0.51)	2 (0.88)	8 (0.40)	6 (0.33)
10	11 (0.29)	4 (0.16)	6 (0.51)	1 (0.44)	9 (0.45)	2 (0.11)
11	5 (0.13)	1 (0.04)	2 (0.17)	2 (0.88)	2 (0.10)	3 (0.16)
12	3 (0.08)	1 (0.04)	2 (0.17)	0 (0.00)	2 (0.10)	1 (0.05)
13	2 (0.05)	1 (0.04)	0 (0.00)	1 (0.44)	1 (0.05)	1 (0.05)
14	1 (0.03)	0 (0.00)	1 (0.09)	0 (0.00)	1 (0.05)	0 (0.00)
15	1 (0.03)	0 (0.00)	1 (0.09)	0 (0.00)	1 (0.05)	0 (0.00)
Mean	1.82 ± 1.98	1.52 ± 0.79	2.25 ± 2.14	2.96 ± 2.31	2.04 ± 2.09	1.59 ± 1.83
<3	2737 (71.52)	1885 (77.48)	737 (63.21)	115 (50.44)	1354 (67.46)	1383 (75.99)
≥3	1090 (28.48)	548 (22.52)	429 (36.79)	113 (49.56)	653 (32.54)	437 (24.01)

Note. CCI = Charlson comorbidity index. Data were presented as n (%) or Mean ± SD.

**Table 3:** Comparison of frequency of comorbidities in different age groups of patients with schizophrenia.

Comorbidity		Total (n = 3827)	Age Groups		
			65–74 (n = 2433)	75–84 (n = 1166)	85+ (n = 228)
Myocardial infarction		66 (1.72)	36 (1.48)	23 (1.97)	7 (3.07)
Congestive heart failure		357 (9.33)	151 (6.21)	161 (13.81)	45 (19.74)
Peripheral vascular disease		114 (2.98)	55 (2.26)	47 (4.03)	12 (5.26)
Cerebrovascular disease		752 (19.65)	401 (16.48)	274 (23.50)	77 (33.77)
Dementia		1294 (33.81)	579 (23.80)	559 (47.94)	156 (68.42)
Chronic pulmonary disease		973 (25.42)	468 (19.24)	395 (33.88)	110 (48.25)
Rheumatic disease		75 (1.96)	46 (1.89)	24 (2.06)	5 (2.19)
Peptic ulcer disease		834 (21.79)	470 (19.32)	299 (25.64)	65 (28.51)
Mild liver disease		442 (11.55)	269 (11.06)	143 (12.26)	30 (13.16)
Diabetes		891 (22.29)	577 (23.72)	265 (22.72)	49 (21.49)
Hemiplegia or paraplegia		53 (1.38)	31 (1.27)	17 (1.46)	5 (2.19)
Renal disease		188 (4.91)	91 (3.74)	71 (6.09)	26 (11.40)
Any malignancy ^a^		169 (4.42)	94 (3.86)	61 (5.23)	14 (6.14)
Moderate or severe liver disease		15 (0.39)	6 (0.25)	7 (0.60)	2 (0.88)
Metastatic solid tumor		25 (0.65)	13 (0.53)	10 (0.86)	2 (0.88)
AIDS/HIV			-	-	-
	65–69	70–74	75–79	80–84	85+
Dementia	276 (18.76)	303 (31.50)	315 (42.74)	244 (56.88)	156 (68.42)
Diabetes without chronic complication	299 (20.33)	170 (17.67)	138 (18.72)	76 (17.72)	39 (17.11)
Diabetes with chronic complication	71 (4.83)	37 (3.85)	35 (4.75)	16 (3.73)	10 (4.39)

Note. Data were presented as: n (%). ^a^: Any malignancy, including lymphoma and leukemia, except malignant neoplasm of skin. AIDS, acquired immunodeficiency syndrome.

**Table 4:** Cox proportional hazards regression analysis for 5-year mortality.

	Univariate Analysis		Multivariable Analysis	
	Hazard Ratio (95% CI)	*p* Value	Hazard Ratio (95% CI)	*p* Value
Age	1.08 (1.08–1.09)	*p* < 0.0001	1.07 (1.06–1.08)	*p* < 0.0001
Sex				
Male	2.09 (1.87–2.34)	*p* < 0.0001	1.66 (1.47–1.87)	*p* < 0.001
Female	1		1	
CCI score	1.20 (1.17–1.23)	*p* < 0.0001	1.14 (1.12–1.17)	*p* < 0.0001
Working type				
Heavy labor	1		1	
Moderate labor	1.31 (1.07–1.59)	*p* = 0.0072	1.25 (1.03–1.52)	*p* = 0.0264
Light labor	1.74 (1.47–2.06)	*p* < 0.0001	1.25 (1.05–1.49)	*p* = 0.0136
Hospital location				
North	1			
Center	1.06 (0.91–1.24)	NS		
South	1.14 (0.99–1.32)	NS		
East and Offshore	1.83 (1.58–2.12)	*p* < 0.0001		
Hospital level				
Medical Centers	1			
Metropolitan H.	0.91 (0.76–1.09)	NS		
Local Community H.	1.63 (1.38–1.92)	*p* < 0.0001		
Physician Clinics	1.14 (0.42–3.07)	NS		

## Data Availability

Data are available from the National Health Insurance (NHI) research database published by the Taiwan NHI administration. Due to the legal restrictions imposed by the government of Taiwan concerning the Personal Information Protection Act, and related regulations, the data cannot be made publicly available.
